# Cadmium-Tolerant Bacterium Strain Cdb8-1 Contributed to the Remediation of Cadmium Pollution through Increasing the Growth and Cadmium Uptake of Chinese Milk Vetch (*Astragalus sinicus* L.) in Cadmium-Polluted Soils

**DOI:** 10.3390/plants13010076

**Published:** 2023-12-26

**Authors:** Bo Wang, Minghui Sun, Yuekai Wang, Tengyue Yan, Yuhang Li, Xinxin Wu, Youbao Wang, Weibing Zhuang

**Affiliations:** 1 School of Ecology and Environment, Anhui Normal University, Wuhu 241002, China; xiaobo20220901@ahnu.edu.cn (B.W.);; 2 Jiangsu Key Laboratory for the Research and Utilization of Plant Resources, Institute of Botany, Jiangsu Province and Chinese Academy of Sciences (Nanjing Botanical Garden Mem. Sun Yat-Sen), Nanjing 210014, Chinaliyuh1998@163.com (Y.L.); 3 School of Life Science and Food Engineering, Huaiyin Institute of Technology, Huaian 223003, China; 11180052@hyit.edu.cn

**Keywords:** antioxidant defense system, *Bacillus nitratireducens*, bioremediation of Cd-contaminated soils, Cd-tolerant bacterium strain Cdb8-1, Chinese milk vetch

## Abstract

Cadmium (Cd) pollution has attracted global attention because it not only jeopardizes soil microbial ecology and crop production, but also threatens human health. As of now, microbe-assisted phytoremediation has proven to be a promising approach for the revegetation of Cd-contaminated soil. Therefore, it is important to find such tolerant microorganisms. In the present study, we inoculated a bacteria strain tolerant to Cd, Cdb8-1, to Cd-contaminated soils and then explored the effects of Cdb8-1 inoculation on the performance of the Chinese milk vetch. The results showed plant height, root length, and fresh and dry weight of Chinese milk vetch grown in Cdb8-1-inoculated soils increased compared to the non-inoculated control group. The inoculation of Cd-contaminated soils with Cdb8-1 also enhanced their antioxidant defense system and decreased the H_2_O_2_ and malondialdehyde (MDA) contents, which alleviated the phytotoxicity of Cd. The inoculation of Cdb8-1 in Cd-contaminated soils attenuated the contents of total and available Cd in the soil and augmented the BCF and TF of Chinese milk vetch, indicating that the combined application of Cd-tolerant bacteria Cdb8-1 and Chinese milk vetch is a potential solution to Cd-contaminated soils.

## 1. Introduction

With the progress of human society and economic development, intense mining and smelting activities as well as the misuse of synthetic pesticides and chemical fertilizers occurred [1,2,3,4], which lead to serious environmental pollution, especially heavy metal pollution, including mainly cadmium, copper, chromium, nickel, lead, and so on [5,6]. In China, the heavy metal contents in 16.1% of the sampling sites are above the allowed threshold, 7.0% of which is Cd (Ministry of Environmental Protection and Ministry of Land and Resources, 2014). Due to its easy migration and transformation, high bioaccumulation, difficult removal, and persistent toxicity [7], excess Cd often jeopardizes plant growth, crop production, and microorganism reproduction in soils [8,9,10]. Cd pollution leads to soil degradation, and also reduces soil productivity, which in turn threatens food quality, human health, and environmental safety. Therefore, there is an urgent need to remediate Cd-contaminated soils, and much more low-cost, environmentally friendly remediation strategies are urgently required.

Several remediation strategies for Cd-contaminated soils have been developed [11]. However, conventional remediation methods, such as physical, chemical, and biological remediation are extremely expensive or mostly ineffective [12]. Compared to the conventional remediation methods, phytoremediation has an advantage in cost and environmental suitability, which allows plants species to extract pollutants from the environment, to immobilize or to turn them into harmless species [13]. However, most plants can not grow well when challenged by heavy metals, which restrict the applications of phytoremediation. Over recent years, microbe-assisted phytoremediation has gained increasing attention because of its environmental suitability and low cost [14]. Microorganisms are easily to be mass cultured at a lower cost and typically have a powerful ability to inactivate or to remove heavy metals [15]. Some heavy metal-resistant microorganisms have been identified for the soil bioremediation [16]. Compared with biochars (1%, *w*/*w*), microorganisms (2%, *v*/*v*) had a better effect to decrease Cd contents in rice grains [17]. Four bacteria have been isolated and have been proven to decrease Cd accumulation of rice when suffering 200 μM of CdCl_2_ [18]. Although numerous reports have documented potential bioremediation by microbial inoculation, much more cost-effective strains are needed in the future. 

The Chinese milk vetch (*Astragalus sinicus* L.) is a globally accepted green manure [19] that has long been employed to increase soil nutrients and crop productivity [20]. Recent studies also documented its potential for the remediation of heavy metal-contaminated soils [21]. For instance, Chinese milk vetch cultivation reduced Cd bioavailability in soil and Cd concentration in rice by reshuffling microbial communities and soil physiochemical properties [22]. Similarly, the Chinese milk vetch growth could turn the exchangeable Cd to its immobile forms, which substantially reduced the bioavailable Cd [23]. Also, the cultivation of Chinese milk vetch has been linked to increased soil organic matter and total nitrogen, which unexpectedly activates soil Cd and primes its uptake by the Napier grass [24]. In contrast, Zheng et al. [25] reported that the Chinese milk vetch inhibited the transfer of Cd, increased the richness of anaerobic microorganisms, and reduced Cd concentration in rice grain. Up to now, few studies have ever explored the remediation effects of cadmium pollution by the integration of Chinese milk vetch and Cd-tolerant bacteria in soil. The present study is to explore the remediation effects of cadmium pollution-combined Chinese milk vetch with Cd-tolerant bacteria Cdb8-1 in soil. Inoculation of different content Cdb8-1 Low (10 mL), medium (20 mL), and high dose group (30 mL) into Cd-contaminated soil, the physiology and biochemistry of Chinese milk vetch were investigated, including some growth parameters, MDA, SOD, POD, and CAT activity. The soil physiochemical properties were also determined in the Cd-contaminated soils remediated by Chinese milk vetch and Cd-tolerant strain Cdb8-1. The contents of Cd, the BCF and TF of Chinese milk vetch, and the soil physiochemical properties after the inoculation of Cdb8-1 were also explored. The following three objectives were addressed in the present study: the effect of Cdb8-1 on (1) growth, (2) the antioxidant defense system of Chinese milk vetch, and (3) Cd concentration and availability in the soil.

## 2. Results

### 2.1. Isolation and Identification of Cd-Tolerant Bacteria

**Figure 1:**
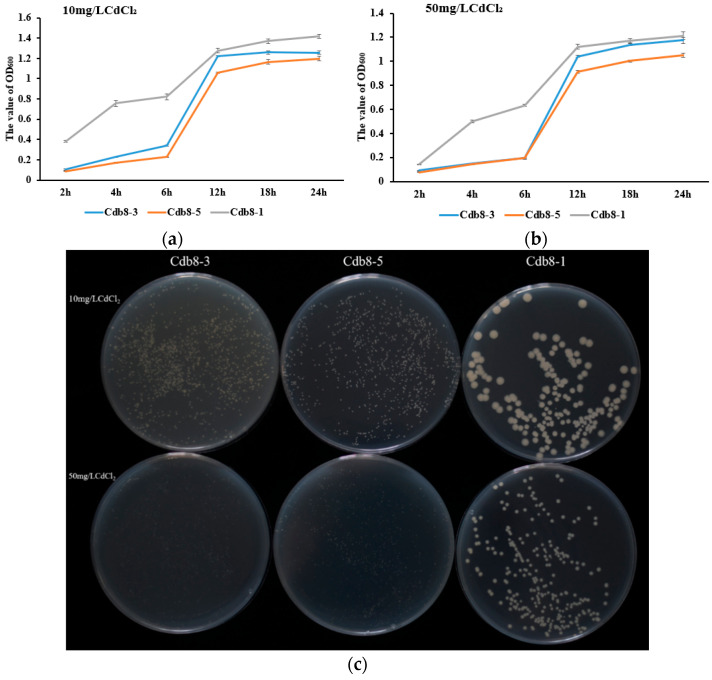
OD600_nm_ value of the Cd-tolerant strains Cdb8-1, Cdb8-3, and Cdb8-5 at several time points on the LB culture medium and LB solid culture plate containing 10 and 50 mg/L CdCl_2_ after 24 h growth. (**a**) 24-hour growth curve of Cdb8-1, Cdb8-3, Cdb8-5 strain in LB liquid medium containing 10 mg/L cadmium chloride (**b**) 24-hour growth curve of Cdb8-1, Cdb8-3, Cdb8-5 strain in LB liquid medium containing 50 mg/L cadmium chloride (**c**) Colony growth status of Cdb8-1, Cdb8-3, Cdb8-5 strain in LB solid medium containing 10mg/L and 50mg/L cadmium chloride.

**Figure 2:**
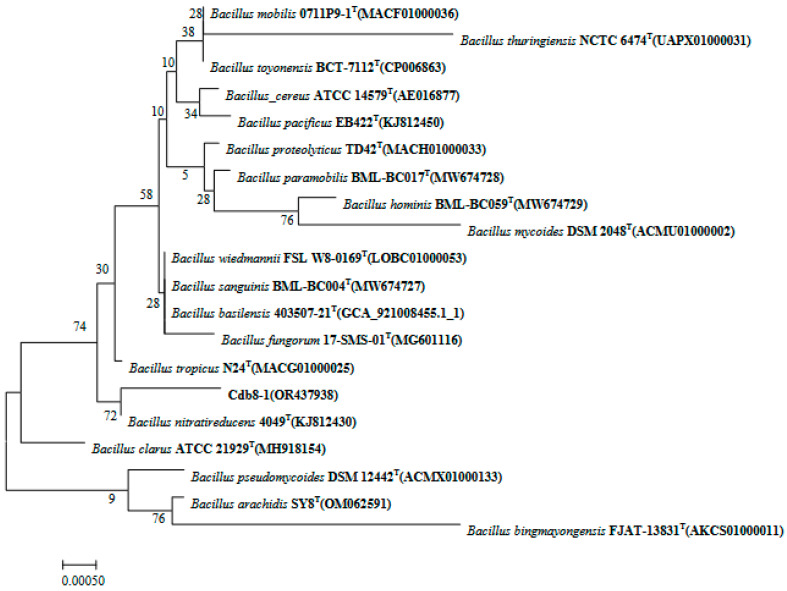
Phylogeny of 16S rRNA sequences. Accession numbers are shown in brackets.

Three Cd-tolerant bacteria were isolated and were designated as Cdb8-1, Cdb8-3, and Cdb8-5. Growth and Cd removal efficiency of these three bacterial strains were tested. As shown in Figure 1, Cdb8-1 propagated significantly faster and better under 10 or 50 mg/LCdCl_2_ compared with Cdb8-3 and Cdb8-5, and therefore was considered as a potential Cd-tolerant bacteria. To identify the bacteria variety of Cdb8-1, 16S rDNA sequences of Cdb8-1 [accession number OR437938 (NCBI)] and other bacteria that showed high homology were used to build the phylogenetic tree by using the neighbor-joining method with a bootstrap value of 1000. The results showed that Cdb8-1 has a 72% sequence similarity to the genus *Bacillus nitratireducens* (Figure 2). Therefore, Cdb8-1 was identified as *Bacillus nitratireducens*.

### 2.2. Effects of Cdb8-1 Inoculation on Chinese Milk Vetch Growth in Cd-Contaminated Soil

**Figure 3:**
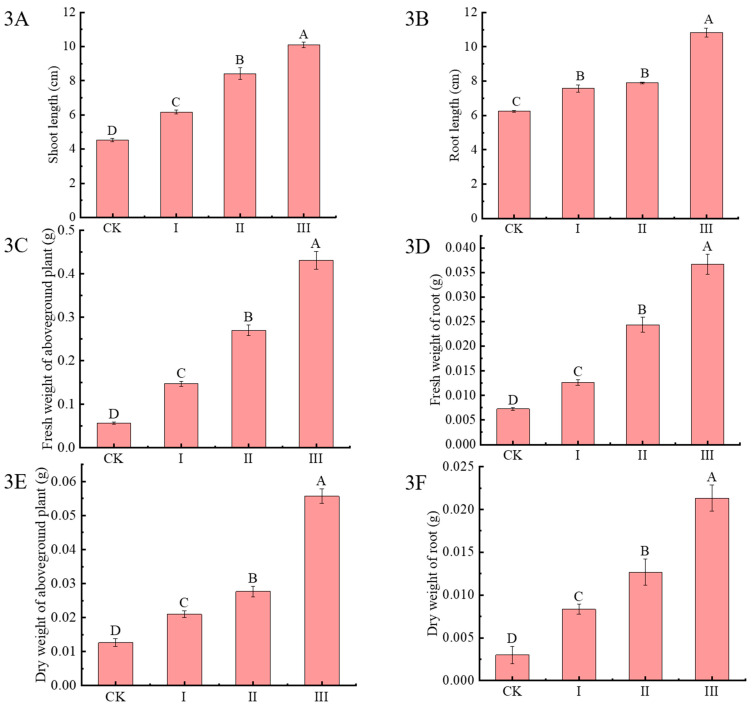
Effects of bacteria inoculation on the growth status of Chinese milk vetch under Cd-contaminated soil. (3**A**): shoot length; (3**B**): root length; (3**C**): fresh weight of shoots; (3**D**): fresh weight of roots; (3**E**): dry weight of shoots; (3**F**): dry weight of roots. Data reported in the figures are means of three replicates (*n* = 3). Duncan’s test was used to do multiple comparison at *p* < 0.05.

The effects of Cdb8-1 on shoot lengths and root lengths of Chinese milk vetch were evaluated after two months (Figure 3). Compared with CK, the shoot lengths and root lengths of Chinese milk vetch increased significantly under Cd-contaminated soil, and the contents of Cdb8-1 are higher, and the shoot lengths and root lengths are much better. There is a 123, 85.9, and 36.2% higher for the shoot lengths and 73.3%, 26.4%, and 21.1% higher for the root lengths under the treatment III, II, and I, respectively, compared with those under CK treatment (Figure 3A,B). Meanwhile, the fresh and dry weights of the above- and under-ground parts increased gradually with the increase of Cdb8 contents (Figure 3C–F). The fresh weights of aboveground parts were 616.7%, 350%, and 150% higher and the fresh weights of underground parts were 404.4%, 234.7%, and 74.3% higher under the treatment III, II, and I, respectively, compared with those under CK treatment (Figure 3C,D). Meanwhile, the dry weights of aboveground parts were 339.4%, 118.4%, and 65.8% higher and those of underground parts were 611%, 322.3%, and 177.7% higher under treatments III, II, and I, respectively, compared with these under treatment CK (Figure 3E,F).

### 2.3. Effects of Cdb8-1 on the Photosynthetic Pigment Contents of Chinese Milk Vetch under Cd-Contaminated Soil

**Figure 4:**
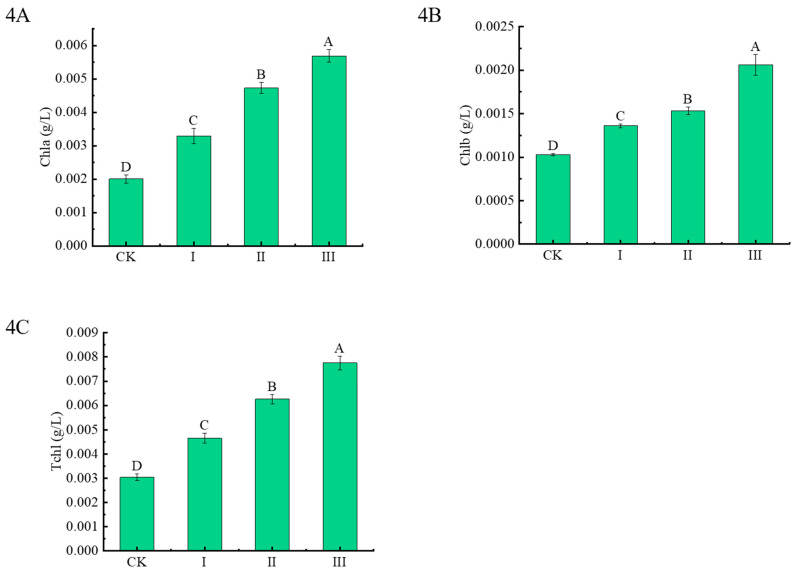
Effects of bacteria inoculation on chlorophyll contents of Chinese milk vetch under Cd-contaminated soil. (4**A**): chlorophyll a contents; (4**B**): chlorophyll b contents; (4**C**): total chlorophyll contents; Data reported in the figures are means of three replicates (*n* = 3). Duncan’s test was used to do multiple comparison at *p* < 0.05.

The contents of photosynthetic pigments are linked to crop health and plant productivity. Compared with CK, the contents of chlorophyll a, b, and total chlorophyll of Chinese milk vetch increased significantly under Cd-contaminated soil, and the contents of Cdb8-1 are higher, the corresponding contents are much better. There is a 183.1%, 135.3%, and 63.7% higher for the chlorophyll a content, 100%, 48.5%, and 32% higher for the chlorophyll b content and 154.9%, 105.9%, and 53% for the root lengths under the treatment III, II, and I, respectively, compared with these under CK treatment (Figure 4A–C). 

### 2.4. Effects of Cdb8-1 Inoculation on the Antioxidant Enzyme System and Contents of H_2_O_2_ and MDA of Chinese Milk Vetch under Cd-Contaminated Soil

**Figure 5:**
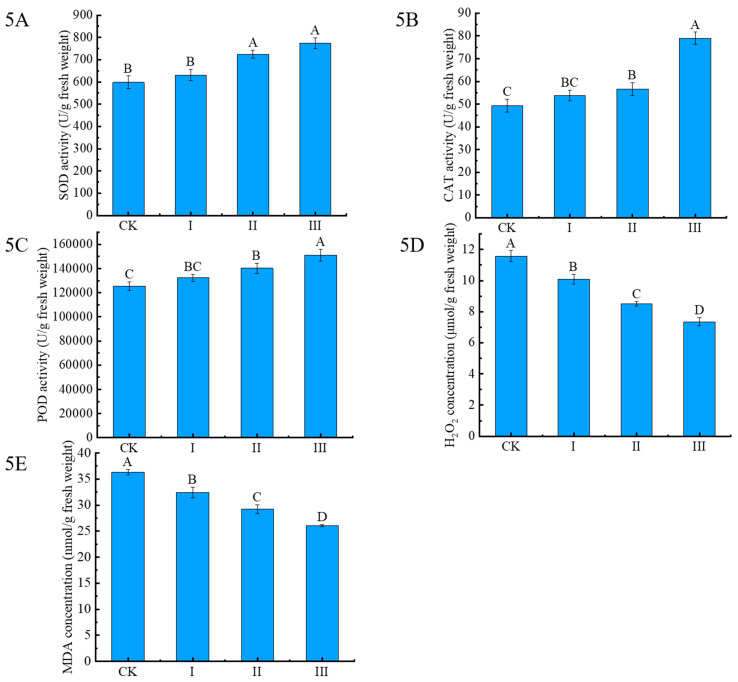
Effects of bacteria inoculation on the antioxidant defense system and oxidative damage of Chinese milk vetch under Cd-contaminated soil. (5**A**): SOD activity; (5**B**): CAT activity; (5**C**): POD activity; (5**D**): content of H_2_O_2_; (5**E**): content of MDA. Data reported in the figures are means of three replicates (*n* = 3). Duncan’s test was used to do multiple comparison at *p* < 0.05.

The effects of Cdb8-1 inoculation on the antioxidant enzyme system and oxidative damage of Chinese milk vetch were evaluated after two months (Figure 5). Compared with the SOD and POD content of Chinese milk vetch under CK treatment, the activities of SOD and POD increased gradually with the increasing dosage of Cdb8-1 (Figure 5). There is a 29.6%, 21.3%, and 5.6% higher for the SOD content and 20.4%, 11.9%, and 5.5% for the POD content under the treatment III, II, and I, respectively, compared with these under CK treatment (Figure 5A,C). However, no significant increase in SOD activity was observed between CK and treatment I, and between treatment II and III. There is also no significant increase of POD content between treatment CK and I, and between treatment I and II. There is a 46.7% and 5.1% higher for the CAT content under the treatment III and II, respectively, compared with these under CK treatment (Figure 5B). Compared with the H_2_O_2_ content and MDA content of Chinese milk vetch under CK treatment, the corresponding content of H_2_O_2_ and MDA decreased gradually with the increase of Cdb8-1 content, although the difference of H_2_O_2_ content between treatment CK and I, and between treatment II and III is not significant (Figure 5D,E). There is a 29.4%, 22.5%, and 2% lower for the H_2_O_2_ content and 28.3%, 19.5%, and 10.7% for the MDA content under the treatment III, II, and I, respectively, compared with those under treatment CK (Figure 5D,E). 

### 2.5. Effects of Cdb8-1 on Cd Uptake by Chinese Milk Vetch under Cd-Contaminated Soil

Compared with the Cd concentration in soil under CK treatment, the Cd content decreased gradually with increasing Cdb8-1 dosage (Table 1). Treatment III had the lowest Cd concentration in soil, and treatment II and I followed. On the contrary, the Cd concentration in the whole plant under treatment III, II, and I is much higher than those under CK treatment, indicating that Cdb8-1 could enhance the Cd uptake in soil via Chinese milk vetch. Interestingly, the Cd concentration in the shoots is much higher than these in roots under CK, I, II, and III, suggesting that Chinese milk vetch is a good choice to conduct the Cadmium remediation. 

The Cd bioconcentration factor of whole plants increased with the increase of Cdb8-1 content, while the transport factors decreased gradually (Table 2). The underground BCF was higher than that of the aboveground one, suggesting that the underground parts of Chinese milk vetch have a higher Cd-enrichment ability. The BCF value of underground parts were >1, and the BCF of aboveground parts and Cd transport factors of Chinese milk vetch was all <1. The lower aboveground BCF and TF and higher underground BCF thus indicate lower translocation ability and higher fixation ability of roots.

### 2.6. Effects of Cdb8-1 on Soil Physiochemical Properties by Chinese Milk Vetch under Cd-Contaminated Soil

Cdb8-1 inoculation has changed many physical and chemical properties of soil. Compared with the soil Cd concentration in CK, the pH, and the content of AN and SOM increased under treatment I, II, and III, and the contents of total and available Cd decreased gradually with the increase of Cdb8-1 content (Table 1 and Table 3). There was no significant difference in other soil physical and chemical properties such as TP, TN, TK, AP, and AK. Cdb8-1 inoculation decreased the content of total Cd and increased the available Cd content in Chinese milk vetch, indicating that Chinese milk vetch combined with Cd-tolerant strain Cdb8-1 could be used as an alternative approach for the remediation of Cd-polluted soil.

### 2.7. Redundant Analysis of TCd, ACd and Physicochemical Factors Respectively

**Figure 6:**
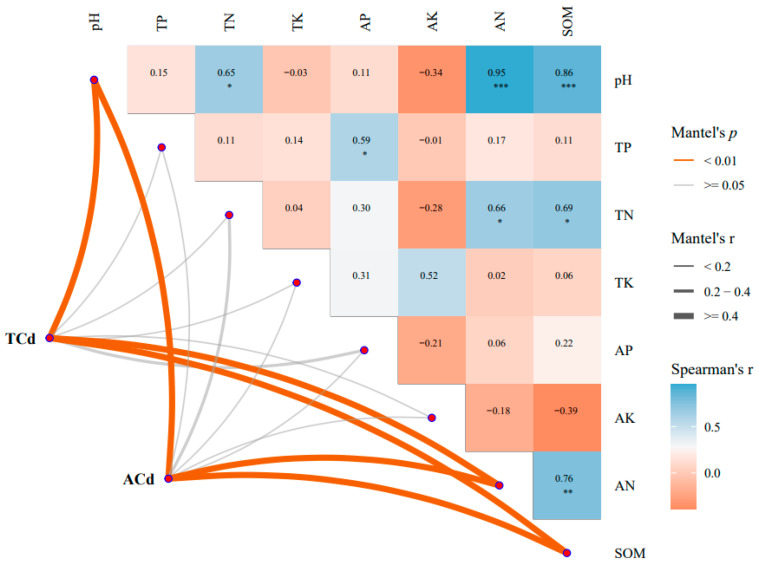
Redundant analysis of TCd, ACd and physicochemical factors, respectively. *, Significantly different representatives ** The difference is very significant *** The difference is extremely significant.

Correlation and RDA analysis showed that TCd and ACd were correlated with pH, AN, SOM physicochemical factors, pH was positively correlated with TN, AN, SOM, TN was positively correlated with SOM, AN was significantly positively correlated, and AN was significantly positively correlated with SOM (Figure 6). 

## 3. Materials and Methods

### 3.1. Materials

The Chinese milk vetch seeds were purchased from Anhui Wuhu Qingyijiang Seed Industry Co., Ltd. (Wuhu, China) The test soil (0–20 cm depth surface soil) was from Laoyaling Tailings Pond, Tianmen Town, Tongling, Anhui Province, China. Impurities in the test soil were removed. The test soil was then air dried and passed through a 2 mm mesh screen before experimentation. Then, each plastic pot was filled with 1600 g of soil. The test soil contains 21.26 g/kg of organic matter, and the pH value is 7.91; the total concentrations of N, P, K, and Cd in the test soil were 1.16 g/kg, 0.50 g/kg, 13.44 g/kg, and 1.77 mg/kg, respectively, while the available N, P, K, and Cd were 94.90 mg/kg, 16.76 mg/kg, 296.88 mg/kg, and 0.69 mg/kg, respectively. 

The bacterium strains were selected from the soil collected from the farmland around the mining site in Qixia District (118.962831E, 32.164459N), Nanjing, Jiangsu Province. The Cdb8-1 was identified as *Bacillus nitratireducens* based on 16Sr RNA analysis and phylogenetic analysis. In our study, the bacterium strain Cdb8-1 could grow well on the LB culture medium containing 50 mg/L Cadmium chloride at most, indicating its tolerance to high level of Cd.

### 3.2. Bacterial 16S rRNA Sequencing and Phylogenetic Analysis

Genomic DNA of the Cdb8-1 was extracted using a genome DNA isolation kit (Tiangen, Beijing, China). Bacterial 16S rRNA sequences were amplified using the universal primers. The forward primer for PCR is 27F (5′-AGAGTTTGATCCTGGCTCAG-3′), and the reverse primer is 1492R (5′-GGTTACCTTGTTACGACTT-3′). The PCR products were sequenced by the BGI Biological Technology Co., Ltd., Shanghai, China, and the resulting sequences were further used to perform taxonomical identification.

Phylogenetic analysis was conducted by using the software MEGA5.1 [26], and aligned using the online software package CLUSTALX8.1 (http://www.clustal.org/clustal2/ accessed on (10 December 2023)). The phylogeny was constructed with the neighbor-joining method at a 1000 bootstrap replications.

### 3.3. Pot Experiments

The experiments included four groups, namely the control group (CK, 30 mL deionized water), the low dose group (I, 10 mL bacterial agent and 20 mL deionized water), the medium dose group (II, 20 mL bacterial agent and 10 mL deionized water), and the high dose group (III, 30 mL bacterial agent), 5.1 × 10^7^ CFU/mL, and each group has five parallel replicates. The specifications of pots were as follows: diameter 16 cm, height 16.5 cm, bottom diameter 12.5 cm. Before sowing the Chinese milk vetch seeds, 500 mL of deionized water was poured into each pot. The 20 seeds of Chinese milk vetch were sown into the pot placed in a greenhouse on April 6 2023. Each group added the corresponding dose once a week for reinforcement. Field management and maintenance, including regular watering and the removing of weeds, are consistent; watering occurs twice a week, with 100 mL each time. After two months, the plants were uprooted; the physio-biochemical indicators including plant height, root length and biomass, the activity of antioxidant enzymes SOD, CAT and POD, and the contents of MDA and H_2_O_2_ of leaf tissues were all measured. The pH, TP, TN, TK, AP, AN, SOM, ACd, and TCd of the soil were also evaluated.

### 3.4. Methods

After two months, plant height, root length, as well as the fresh and dry weights of the Chinese milk vetch were measured. For dry weight measurement, the plants were dried in an oven at 105 °C for 30 min and then at 80 °C until no weight loss [27]. The photosynthetic pigments were extracted using 80% acetone and then determined using a colorimetric method [28]. The contents of MDA and H_2_O_2_ and the activities of antioxidant enzymes, superoxide dismutase (SOD), peroxidase (POD), and catalase (CAT) were measured based on previous approaches [29,30,31,32,33]. For soil pH determination, one gram of soil was extracted using 2.5 mL distilled water, and the pH value was determined using a pH meter (PHS–3C, Shanghai Dapu Instruments, Shanghai, China). Soil organic matter (SOM) and total N (TN) were determined with an elemental analyzer (Vario MACRO cube, Elementar, Germany). The total K (TK) was extracted using NaOH and the available potassium (AK) was extracted by using ammonium acetate, and both contents was determined with the flame photometry [34]. The total P (TP) was extracted using hydrofluoric and perchloric acid and its content was determined by using the colorimetric method [35]. The available P (AP) was extracted using NaHCO_3_ solution, and AP content was determined using a spectrophotometer (UV-2100UV, Rayleigh, Beijing, China). Total Cd in the soil was extracted using a mixture of HCl, HNO_3,_ and HClO_4_, while the total content of Cd in plant samples were extracted using the mixture of HNO_3_ and HClO_4_ [36]. Different chemical fractions of Cd in the soil was extracted using the Tessier multistage continuous extraction method [37], and Cd concentrations were determined with the atomic absorption spectrophotometer [38]. The available nitrogen was extracted and determined based on the standard protocol as described by Pan et al. [39]. 

### 3.5. Data Analysis

Raw data of the experiment were processed with Microsoft Excel 2021 (Microsoft Inc., Redmond, WA, USA). The data were analyzed using the SPSS 19.0 software package (SPSS Inc., Chicago, IL, USA). One-way analysis of variance (ANOVA) was carried out to test the significance of treatments at *p* < 0.05, followed by Duncan’s tests. The data reported in the paper were the means of three replicates and were expressed as means ± standard errors (*n* = 3).

BCF (bioconcentration factor) = Cd concentration in plants/Cd concentration in soil.

TF (translocation factor) = Cd concentration in shoots/Cd concentration in roots [40]. 

## 4. Discussion

### 4.1. Isolation and Characterization of Cd-Tolerant Bacteria

In this study, the heavy metal-resistant bacteria isolated from the heavy metal-contaminated soil could grow well in solid and liquid media containing high concentrations of CdCl_2_ after three consecutive enrichment screenings, showing a high tolerance and resistance level to heavy metals. In order to clarify the species relationship, the current widely used 16S rRNA identification method was used to provide potential bacterial species for its subsequent use in the remediation of heavy metal-contaminated soil.

### 4.2. Cd-Tolerant Bacteria Cdb8-1 Promote Chinese Milk Vetch Growth in Cd-Contaminated Soil

Plant growth is usually negatively affected by Cd stress, which is probably linked to DNA damage, lipid peroxidation, metabolism dysfunction, photosynthesis reduction, and impaired plant morphology and physiology [41]. Many Cd-tolerant bacteria have been identified to promote plant growth and enhance the phytoremediation of heavy metal-contaminated soils [42,43]. In the present study, three Cd-tolerant bacteria were identified, and the strain Cdb8-1 was most resistant to Cd in culture and can be considered as a Cd-tolerant bacteria. Cd-tolerant bacteria Cdb8-1 can promote the growth of Chinese milk vetch under Cd-contaminated soil significantly, compared with the CK, including the shoot and root lengths, as well as the fresh and dry weights of the aboveground parts. Many Cd-tolerant plant growth-promoting bacteria have been proven effective in increasing plant growth, such as plant height, root lengths, plant biomass, and so on. *Burkholderia gladioli* and *Pseudomonas aeruginosa* increased the biomass of tomato (*Solanum lycopersicum*) grown in Cd-contaminated soil [44]. A Cd-tolerant bacteria strain, Cdq4-2 (*Enterococcus* sp.), enhanced the biomass of *L. perenne* under the Cd-contaminated soil [38]. 4*Pseudomonas* sp. RJ10 and *Bacillus sp*. RJ16 also contributed to the root growth of *B. napus* and increased the root length [45]. Therefore, the enhanced growth of Chinese milk vetch in Cd-contaminated soil inoculated with the Cd-tolerant bacteria Cdb8-1 in this study probably has attenuated the detrimental effects of exogenous Cd application, indicating that the inoculation of Cd-tolerant bacteria Cdb8-1 has facilitated the remediation of Cd-contaminated soil via the Chinese milk vetch.

### 4.3. Effects of Cdb8-1 on the Chlorophyll Content of Chinese Milk Vetch under Cd-Contaminated Soil

The contents of chlorophyll a, chlorophyll b, and total chlorophyll of Chinese milk increased. The chlorophyll plays important roles for plant growth and development. Bashir et al. [46] reported that soil amendments have contributed to the increase of chlorophyll contents and photosynthetic rate of wheat in response to Cd pollution, which increases the amount of dry matter of plants. Sulla coronaria inoculated with rhizobacteria increased their chlorophyll content under the Cd-contaminated soil compared with the control plants, and Mishra et al. [47] also observed that chlorophyll content and leghemoglobin of pea were increased in response *Pseudomonas* strain PGERs17 and *Rhizobium leguminosarum* inoculation. Cd-tolerant bacteria Cdb8-1 alleviated the phytotoxicity of Cd through the regulation of antioxidant defense system in Chinese milk vetch.

### 4.4. Effects of Cdb8-1 on the Antioxidant Defence System and Oxidative Damage of Chinese Milk Vetch under Cd-Contaminated Soil

The reactive oxygen species (ROS) generation is a general response of living organisms to abiotic stress, and the oxidative stress occurred when they were produced in large amounts [48]. To avoid or alleviate the oxidative stress, plants have developed a sophisticated antioxidant defense systems, including mainly superoxide dismutase (SOD), POD, catalase (CAT), ascorbate peroxidase (APX), and peroxidase (POX), such as guaiacol peroxidase (GPOX). The primary deleterious effect of Cd mainly includes the induction of oxidative stress and breakage of plant cell membrane through the generation of ROS. SOD, POD, and CAT are three critical antioxidant enzymes to quench ROS, thereby preventing cell membrane peroxidation, and oxidative injuries [49]. In our study, the Cd-tolerant bacteria Cdb8-1 probably has alleviated the toxic effects of Cd by upregulating antioxidant defense system in Chinese milk vetch.

SOD can effectively remove the ROS, which can convert O_2_ to H_2_O_2_ [50]. A previous study has shown that Cd stress increased SOD activity in *L. perenne*, and the increase of SOD activity functions to alleviate the toxic effects of Cd on *L. perenne* by removing the excess ROS [38,51]. There was a significant increase in SOD activity in soil inoculated with wheat leaves, while actinomycetes application could further enhance SOD activity [52]. Lactobacillus plantarum/acidophilus/rhamnosus treatment also promoted the activity of SOD in leaves of *Brassica juncea* compared to control [53]. In agreement with previous researches, the SOD activities increased gradually with the increase of Cdb8-1 content, which has alleviated the phytotoxicity of Cd to some extent. 

POD is an enzyme closely linked to plant respiration and photosynthesis [54] that can be used as an indicator of metabolism when plants suffer from various stresses, including Cd stress, drought, salt stress, and so on [53]. Results of the present study showed that POD activity in Chinese milk vetch under Cd-contaminated soil increased significantly after inoculation with Cdb8-1. Similar results were also reported in the research work of Zhu et al. [55] wherein that inoculation of endophytic bacteria has enhanced POD activity in tomatoes. There is an increase of POD activity in combined treatment (*Lactobacillus plantarum*/rhamnosus) compared to the control single treatment (*Lactobacillus plantarum*) reached 15.7%, indicating a positive effect between *Streptomyces pactum* and antioxidant enzymes. These results suggested that Cdb8-1 probably has started the genes responsible for POD enzymes, which in turn alleviates the oxidative damage.

SOD can transform O_2_^−^ radicals into H_2_O_2_, which can be promoted with the increase of APX and CAT activity. The SOD combined with APX and CAT constituted an intrinsic antioxidant defense system to deal with various adversity, including Cd stress. 

In the present study, the CAT content under treatments III and II was found to be 46.7% and 5.1% higher, respectively, compared with those under CK treatment (Figure 5B), and the H_2_O_2_ content decreased gradually with the increase of Cdb8-1 content. In agreement with our results, there is a 1.24-fold increase of H_2_O_2_ content in response to the co-application of Lactobacillus plantarum/acidophilus/rhamnosus compared with the control. The microorganism–plant interactions can enhance the activity of CAT compared with the single treatment, which help plants to better alleviate abiotic stress [56,57].

The ROS-scavenging system, mainly including SOD, POD, CAT, and other antioxidants, functions to remove the excess ROS in plants, maintaining cellular homeostasis [58,59]. Microorganisms used antioxidant defense systems to avoid the damage when they suffer from high concentrations of heavy metals [60]. Therefore, Chinese milk vetch applied their antioxidant defense system through upregulating the antioxidant enzyme activities to prevent oxidative damage under Cd-contaminated soil, which was enhanced by the Cd-tolerant strain Cdb8-1.

MDA (malondialdehyde), a widely used indicator of oxidative stress, is the product of membrane lipid peroxidation [61]. In response to excess Cd exposure, plants produce more ROS than the capacity of ROS-scavenging system, leading to oxidative damage and large amounts of MDA; the content of MDA is negatively associated with their antioxidant defense system [62]. Previous studies show that Cd treatment increased the activity of SOD, but decreased MDA content in rice [63]. The concentration of MDA in *L. perenne* was also decreased to some extent under Cd contamination soil [38]. In the present study, MDA content in Chinese milk vetch leaves decreased gradually with the increase of Cdb8-1 content under Cd-contaminated soil. Zhang et al. [64] also found a negative correlation between MDA content and SOD activity, which is consistent with our results. Previous studies indicated that lower MDA content is positively correlated to higher heavy metal tolerance, which also suggested that Chinese milk vetch is tolerant to Cd stress [38,65]. The above results suggested that plants tolerate Cd stress not only on a single enzyme, but on the entire antioxidant defense system.

### 4.5. Effects of Cdb8-1 Inoculation on Cd Uptake by Chinese Milk Vetch and Soil Physio-Chemical Properties 

The treatment of soil cadmium pollution follows the principle of reducing the total amount and reducing the biological effectiveness. In this study, the total cadmium content decreased gradually with the increase of Cdb8-1 inoculation (Table 1), and the total cadmium content in the soil under treatment III decreased the most, and the level of difference between treatments I, II, and CK reached significant levels, indicating that the interaction between this strain and milk vetch has the ability and application potential to remediate cadmium contaminated soil. A more valuable finding is that the cadmium content in the underground part of the milk vetch plant was much higher than that in the aboveground part of the experimental group and the control group, indicating that milk vetch can be used as a candidate plant material for cadmium pollution remediation. In addition, with the increase of Cdb8-1 inoculation, cadmium enrichment (BCF) increased while the transfer factor (TF) decreased (Table 2). The underground bioenrichment factor was higher than that of the aboveground bioenrichment factor, which once again proved that the underground part of milk vetch had strong cadmium enrichment ability, cadmium pollution remediation ability, and application value.

### 4.6. Effects of Cdb8-1 on Physical and Chemical Properties of the Soil by Chinese Milk Vetch in Cd-Contaminated Soil

Soil fertility, pH value, and other physiochemical properties are important factors influencing plant growth and development. Among these factors, soil pH is closely related to the distribution, transformation, and bioavailability of heavy metals in soil [66]. Previous studies have shown that lower pH may increase the bioavailability of Cd, while higher pH value may inactivate soil Cd [67,68]. Many studies have shown that the inoculation of heavy metal-immobilizing bacteria has increased soil pH, and as a consequence, heavy metal availability was reduced [69]. Therefore, the bioavailability of soil Cd is negatively correlated to pH value [22], and the elevated pH value has likely contributed to the decreased available fraction of Cd in the [70,71,72,73]. In this study, Cd-tolerant bacteria Cdb8-1 inoculation significantly increased soil pH value (Table 3) but decreased the content of the total and available Cd, which is consistent with previous studies. Therefore, the passivation effect of Cd-tolerant bacteria has caused an increase of soil pH value, which further inactivates soil Cd, leading to a cascading passivation of soil Cd. In addition, the results of our study also indicated that the inoculation of Cd-tolerant bacteria has a significant effect on the content of soil AN and SOM. 

On the one hand, the Cd-tolerant bacteria Cdb8-1 inoculation could enhance the growth and development of Chinese milk vetch in Cd-contaminated soil by altering the soil physio-chemical properties. On the other hand, the Cd-tolerant bacteria Cdb8-1 itself could also produce a large quantity of low-molecular-weight organic acids, amino acids, and enzymes, which also could increase the biomass of the Chinese milk vetch. Coincidently, the contents of total and available Cd decreased in the Cd-contaminated soil, but increased in the whole plants, indicating that the combination of Chinese milk vetch with Cd-tolerant bacteria Cdb8-1 can be used to remediate the Cd-contaminated soil. The results indicated that the combination of Chinese milk vetch with Cd-tolerant bacteria Cdb8-1 is a good approach for the remediation of Cd-contaminated soil.

### 4.7. RDA Analysis

ACd is closely related to PH size, and the higher the PH, the smaller the ACd content. PH was significantly positively correlated with TN, AN, SOM, and TN was significantly positively correlated with SOM and AN. TCd was correlated with soil SOM content, and SOM was significantly positively correlated with AN, and RDA analysis showed that pH, AN, and SOM were the main physicochemical factors affecting TCd and ACd.

## 5. Conclusions

Cd-tolerant bacterial inoculation of the soil can improve Chinese milk vetch antioxidant defense system (including SOD, POD, and CAT), reduce the toxic effects of Cd, enhance pollution resistance, and alleviate membrane lipid peroxidation damage and, meanwhile, promoted the growth of Chinese milk vetch. The Total Cd and available Cd concentration in the Cd-contaminated soil was decreased, which is conducive to the remediation of Cd by Cd-tolerant bacterial and Chinese milk vetch. Inoculation also decreased the Cd transport coefficient of Chinese milk vetch, thereby increasing its remediation effect on Cd-contaminated soil.

## Figures and Tables

**Table 1:** Effects of Cdb8-1 on Cd concentration in the soil, aboveground parts, underground parts, and the whole plant of Chinese milk vetch under Cd-contaminated soil. Capital letters represent significant differences.

Treatment	Cd Content in Soil (mg/kg)	Cd Content in the Aboveground Parts (mg/kg)	Cd Content in the Underground Parts (mg/kg)	Cd Content in the Whole Plant (mg/kg)
CK	1.65 ± 0.03 A	0.31 ± 0.01 A	1.92 ± 0.12 C	2.23 ± 0.12 B
I	1.44 ± 0.02 B	0.29 ± 0.02 A	2.87 ± 0.15 B	3.16 ± 0.16 A
II	1.35 ± 0.04 C	0.26 ± 0.03 A	3.18 ± 0.21 AB	3.44 ± 0.18 A
III	1.23 ± 0.02 D	0.17 ± 0.01 B	3.34 ± 0.05 A	3.52 ± 0.04 A

**Table 2:** BCF and TF of Cd in Chinese milk vetch under Cd-contaminated soil. Capital letters represent significant differences.

Treatment	Bioconcentration Factor (BCF)			Translocation Factor (TF)
	Aboveground Parts	Underground Parts	Whole Plant	
CK	0.19 ± 0.01 A	1.17 ± 0.05 D	1.35 ± 0.05 D	0.16 ± 0.01 A
I	0.20 ± 0.02 A	2.00 ± 0.13 C	2.20 ± 0.14 C	0.10 ± 0.00 B
II	0.19 ± 0.02 A	2.35 ± 0.14 B	2.54 ± 0.13 B	0.08 ± 0.01 B
II	0.14 ± 0.01 B	2.72 ± 0.08 A	2.86 ± 0.07 A	0.05 ± 0.00 C

**Table 3:** Effects of Cdb8-1 with different treatment (I, II, and III) on physical and chemical properties of the soil by Chinese milk vetch under Cd-contaminated soil. Capital letters represent significant differences.

	PH	TP (g/kg)	TN (g/kg)	TK (g/kg)	AP (mg/kg)	AK (mg/kg)	AN (mg/kg)	SOM (g/kg)	ACd (mg/kg)	TCd (mg/kg)
CK	8.01 ± 0.02 B	0.61 ± 0.06 AB	1.24 ± 0.08 A	11.91 ± 0.21 A	19.20 ± 2.13 A	243.00 ± 14.27 A	115.77 ± 4.58 C	18.72 ± 0.53 B	0.65 ± 0.04 A	1.65 ± 0.03 A
I	8.07 ± 0.03 B	0.60 ± 0.02 AB	1.20 ± 0.10 A	12.15 ± 0.61 A	18.28 ± 0.73 A	242.67 ± 14.70 A	122.42 ± 3.36 BC	19.05 ± 0.14 B	0.58 ± 0.01 B	1.44 ± 0.02 B
II	8.17 ± 0.03 A	0.53 ± 0.01 B	1.30 ± 0.07 A	11.89 ± 0.17 A	18.02 ± 0.30 A	228.52 ± 14.58 A	134.26 ± 5.73 B	20.24 ± 0.27 A	0.55 ± 0.01 BC	1.35 ± 0.04 C
III	8.23 ± 0.03 A	0.67 ± 0.06 A	1.33 ± 0.01 A	11.76 ± 0.36 A	19.31 ± 0.43 A	228.48 ± 14.46 A	153.76 ± 7.40 A	20.49 ± 0.41 A	0.50 ± 0.01 C	1.23 ± 0.02 D

## Data Availability

Data will be made available on request.

## Environmental Implication

Sustainable resource utilization is a presupposition for the development of modern society. A pot experiment was conducted to explore the effects of different concentrations of Cdb8-1 inoculation on the growth performance of the Chinese milk vetch in Cd-contaminated soils. The results showed that the inoculation of Cd-contaminated soils with Cdb8-1 has increased Chinese milk vetch growth compared with the non-inoculation control group. The inoculation of Cd-contaminated soils with Cdb8-1 has decreased the contents of total and available Cd in the soil and improved the BCF and TF of Chinese milk vetch, indicating that Cd-tolerant bacteria Cdb8-1 combined with Chinese milk vetch is a promising approach for the bioremediation of Cd-contaminated soils.
